# Gamma-ray emission from wakefield-accelerated electrons wiggling in a laser field

**DOI:** 10.1038/s41598-019-38777-3

**Published:** 2019-02-21

**Authors:** Jie Feng, Yifei Li, Jinguang Wang, Dazhang Li, Fang Li, Wenchao Yan, Weimin Wang, Liming Chen

**Affiliations:** 1 Beijing National Research Center of Condensed Matter Physics, Institute of Physics, CAS, Beijing, 100190 China; 2 University of Chinese Academy of Sciences, Beijing, China; 3 Key Laboratory for Laser Plasmas (MoE) and Department of Physics and Astronomy, Shanghai Jiao Tong University, Shanghai, 200240 China; 4 Institute of High Energy Physics, CAS, Beijing, 100049 China; 5 The National Astronomical Observatories, CAS, Beijing, 100012 China; 6 Institute of Physics ASCR, v.v.i. (FZU), ELI BEAMLINES, Za Radnicí 835, Dolni Brezany, 252241 Czech Republic; 7 Songshan Lake Materials Laboratory, Dongguan, Guangdong 523808 China

## Abstract

Ultra-fast synchrotron radiation emission can arise from the transverse betatron motion of an electron in a laser plasma wakefield, and the radiation spectral peak is limited to tens of keV. Here, we present a new method for achieving high-energy radiation via accelerated electrons wiggling in an additional laser field whose intensity is one order of magnitude higher than that for the self-generated transverse field of the bubble, resulting in an equivalent wiggler strength parameter K increase of approximately twenty times. By calculating synchrotron radiation, we acquired a peak brightness for the case of the laser wiggler field of 1.2 × 10^23^ ph/s/mrad^2^/mm^2^/0.1%BW at 1 MeV. Such a high brilliance and ultra-fast gamma-ray source could be applied to time-resolved probing of dense materials and the production of medical radioisotopes.

## Introduction

Ultra-fast X-/γ-ray sources have been widely used to resolve the structure and dynamics of dense matter and biological proteins^1,2^. However, these sources with high brightness are mainly achieved at large facilities, such as X-ray free electron laser (X-FEL)^3^ and synchrotron radiation light sources^4^, which are accessible to a limited number of users. Both of these sources consist of a series of electron acceleration RF cavities with a maximum electrical breakdown field of approximately 100 MV/m and a periodic magnetic structure that forces the electrons to oscillate and emit radiation. In contrast, laser-driven plasma accelerators can generate one thousand times larger acceleration gradients^5–7^, thus reducing a hundred metre synchrotron facility to a few centimetres^8,9^.

Laser wakefield accelerators (LWFAs) have become increasingly mature and have recently exhibited stability^10^, energy tunability^11,12^, femtosecond durations^13^, and low divergence (mrad)^14^ electron bunches with a charge at the pC level^15,16^. When a fraction of background electrons is captured by the electrostatic fields produced by the separation of electrons and ions inside the cavity, these electrons can be accelerated by the longitudinal electric wakefield and undergo transverse oscillations in the ion cavity. The oscillation occurs at the betatron frequency ${\omega }_{\beta }={\omega }_{pe}/\sqrt{2\gamma }$, where ${\omega }_{pe}=\sqrt{4\pi {n}_{e}{e}^{2}/{m}_{e}}$ is the plasma frequency and γ is the relativistic Lorentz factor^17^. The betatron wavelength ${\lambda }_{\beta }=2\pi c/{\omega }_{\beta }$ (~100 μm) is three orders of magnitude smaller than the period of the magnetic structure of the synchrotron light source, and the betatron radiation pulse duration is predicted to be as low as tens of fs. However, the spectral peaks of this radiation are limited to between several and tens of keV^18–20^ because of the electron energy, oscillation amplitude, and frequency^21–23^. For a small oscillation amplitude, the strength parameter $K=\gamma {r}_{\beta }{\omega }_{\beta }/c\ll 1$ is in the undulator limit, and the corresponding radiation spectrum is narrowly peaked at the fundamental energy. As $K\to 1$, radiation also occurs at harmonics. When $K\gg 1$ (the wiggler limit), a broad spectrum of synchrotron radiation is emitted with critical energy ${E}_{crit}\approx 3\hslash {\omega }_{p}^{2}{r}_{\beta }{\gamma }^{2}/2c$ ^24^.

In recent years, much progress has been made in enhancing the betatron radiation exhibited in LWFAs. In 2004, Rousse *et al*.^21^ observed ultra-short pulse X-rays with a peak energy of several keV and a brilliance of 2 × 10^22^ ph/s/mrad^2^/mm^2^/0.1%BW driven by a 1 J, 30 fs laser facility. Kneip *et al*.^17^ showed an enhanced electron energy and emission of higher energy radiation with *E*_*crit*_ = 29 keV by a 2.3 J, 32 fs laser facility. To further increase the radiation energy, K. Huang *et al*.^25^ enhanced the electron oscillation amplitude by letting ionization-injected electrons resonate weakly with the laser-driven pulse and emit hard X-rays with *E*_*crit*_ = 75 keV. In addition, Cipiccia *et al*.^26^ demonstrated betatron radiation with *E*_*crit*_ = 450 keV and emission with a peak brilliance of 10^23^ ph/s/mrad^2^/mm^2^/0.1%BW from self-injected electrons resonating strongly with the laser-driven pulse with a higher energy of 5 J and a longer pulse duration of 55 fs. The last two teams utilized the dephasing of the accelerated electrons with respect to the wakefield. As the electrons catch up with the tail of the laser-driven pulse, enhancement of the oscillation amplitude occurs at resonance with the laser field. However, the electrons entering the laser field will decelerate in the propagation direction, which causes a drop in *E*_*crit*_. Furthermore, the laser intensity has already been seriously decreased by energy depletion, which limits the effectiveness of the enhancement. Only one exception exists: when the laser pulse length is sufficiently long to cover the acceleration field. However, a longer laser pulse and a constant laser energy mean a smaller laser intensity, which results in a smaller electron energy gain $({\rm{\Delta }}E\propto \frac{2}{3}{a}_{0}\frac{{\omega }_{0}^{2}}{{\omega }_{p}^{2}})$^27^. Therefore, in this letter, we propose a new method in which two ultra-short laser pulses drive electron acceleration and betatron radiation independently, which not only can sustain electrons located in the acceleration wakefield to ensure a high energy gain but also induces strong betatron oscillations in the wiggling laser field.

## Results

**Figure 1:**
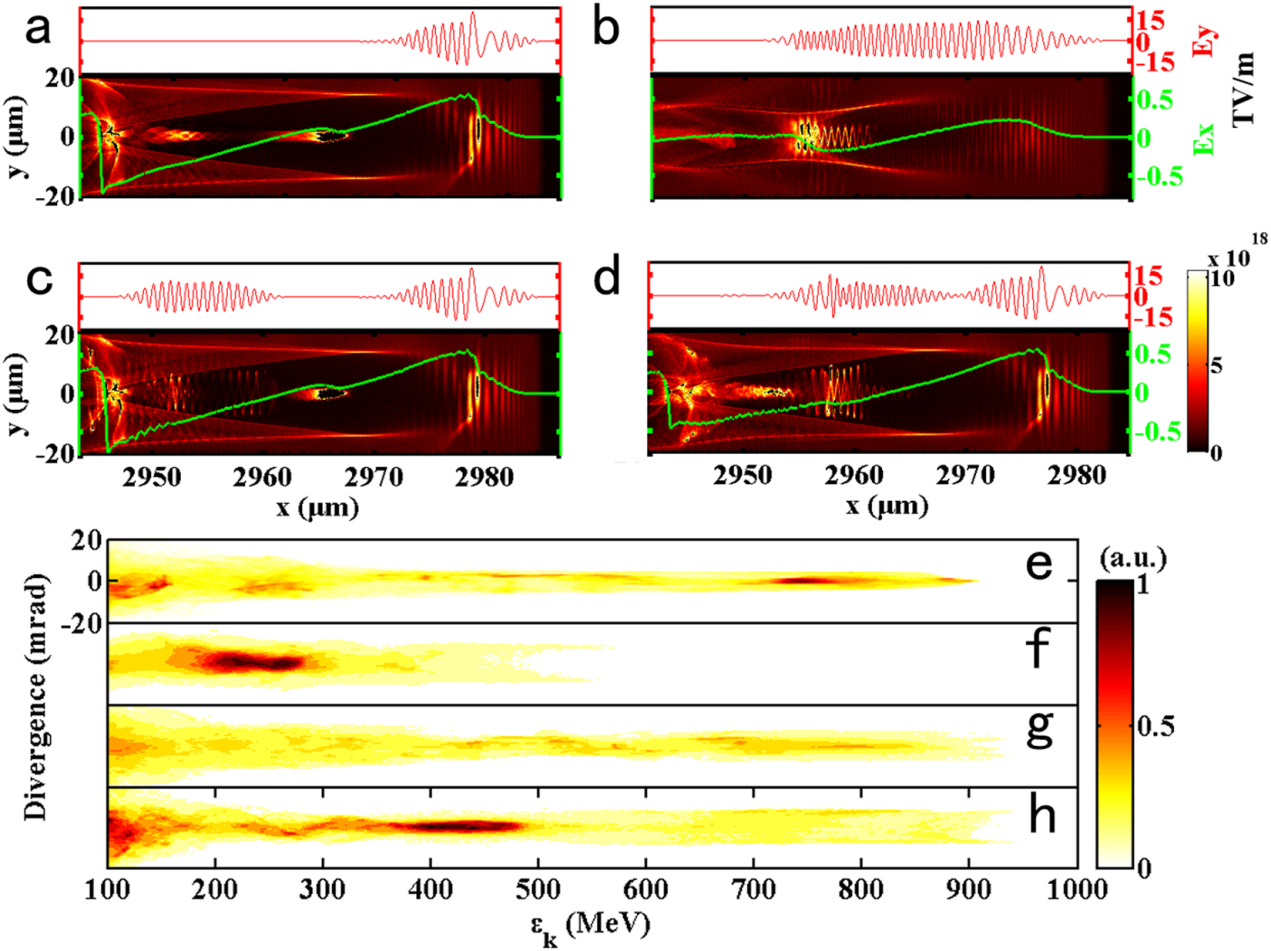
Electron acceleration in four different cases. (**a**,**b**) are snapshots of the LWFA process at t = 10 ps for the cases of the single laser pulse with a laser energy of 3 J and a pulse duration of 30 fs and with values of 4.5 J and 60 fs, respectively. (**c**,**d**) are snapshots of the cases of the two laser pulses with intervals of 120 fs and 60 fs, respectively, with a 3 J, 30 fs driving laser pulse and a 1.5 J, 30 fs second laser pulse. (**e**,**f**) are electron spectra for the four cases.

To compare the results of electron acceleration with or without a second laser pulse as well as two laser pulses with different time intervals, simulations were carried out (see Methods), and the results are shown in Fig. 1(a–h). Because the peak intensity of the driving laser varied with the periodic self-focusing and defocusing and the erosion of the laser pulse front led to self-steeping, as the red line *E*_*y*_ shows in Fig. 1(a,c,d), the fusion of the first bubble and the second bubble was stimulated, forming a large bubble with a length of ~40 μm^16,28^. However, stimulation of this effect by the longer pulse with a lower laser intensity was difficult, and the bubble length was approximately equal to the plasma wavelength ${\lambda }_{p}=2\pi c/{\omega }_{pe}$ ~20 μm, as shown in Fig. 1(b), thus making the bubble shorter than the laser pulse. In this case, the ionization-injected electrons experienced the laser field and were forced to oscillate with an amplitude of ~5 μm, but because of the shorter dephasing length, the maximum electron energy of ~550 MeV was much lower than that in the case with the bubble fusion effect, as shown in Fig. 1(e,f). Meanwhile, the high-energy sections of the energy spectra in 1(a), 1(c) and 1(d) showed a bifurcation structure due to the mechanism of direct laser acceleration (DLA)^29^. To acquire a high-energy electron beam with a larger oscillation amplitude, we proposed a modification based on case (a) corresponding to Fig. 1(a) in which a second intense laser pulse follows the driving laser pulse at different positions of the bubble, as shown in Fig. 1(c,d). A majority of ionization-injected electrons were located in both the second laser pulse and the acceleration field, as shown by the green line, and possessed a large transverse oscillation amplitude relative to case (a). The maximum energy of the electron for the two laser case was slightly larger than that for the single laser case due to the DLA, and the divergence of the high-energy section (>500 MeV) was also larger, especially for case (d) corresponding to Fig. 1(d), as shown in Fig. 1(e,g,h).

**Figure 2:**
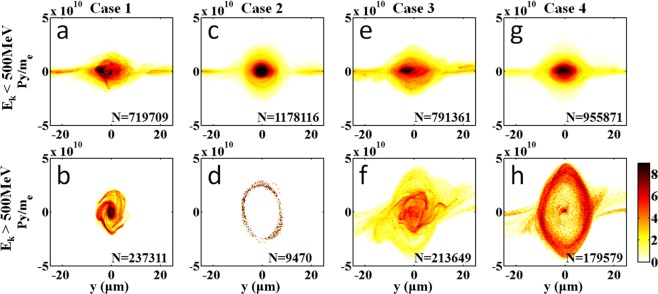
Electron beam phase space of y-py. (**a**,**b**) correspond to the cases of the single laser pulse with electron energy below and above 500 MeV, respectively. (**c**,**d**) correspond to the single laser pulse with a duration of 60 fs. (**e**,**f**) correspond to the two laser case with an interval of 120 fs. (**g**,**h**) correspond to the interval of 60 fs. The numerical values in the figures represent the total number of macroparticles.

To investigate the oscillation behaviour of all accelerated electrons in different cases, it is necessary to compare the different phase spaces (*y, p*_*y*_) of electrons, as shown in Fig. 2. If these electrons have a closed annulus or a circular distribution, these electrons can be more stable in their respective rings. In other words, these electrons have a more stable oscillation in the bubble. Figure 2(a,c,e,g) show a circular distribution accompanied by two arms. This result indicated that the majority of electrons with energy below 500 MeV could oscillate stably in the bubble with amplitudes <5 μm, but a portion of electrons with amplitude >10 μm oscillated out of the stable region. For an electron energy above 500 MeV, the majority of electrons for the two laser pulses with an interval of 120 fs were located in the circle with *r* ~ 6 μm because of the high-energy segment electrons injected outside of the second laser pulse. In contrast, when the interval was 60 fs, the majority of electrons were located in an annulus with 7 μm < *r* < 10 μm, as shown in Fig. 2(h), indicating that the interval of 60 fs was suitable for obtaining an electron beam with higher energy and a larger oscillation amplitude. Moreover, for the single laser pulse case, the amplitudes of most of the electrons (>500 MeV) were less than 2 μm, and the transverse momentum was approximately one-quarter of that in case 4, as shown in Fig. 2(b,h).

**Figure 3:**
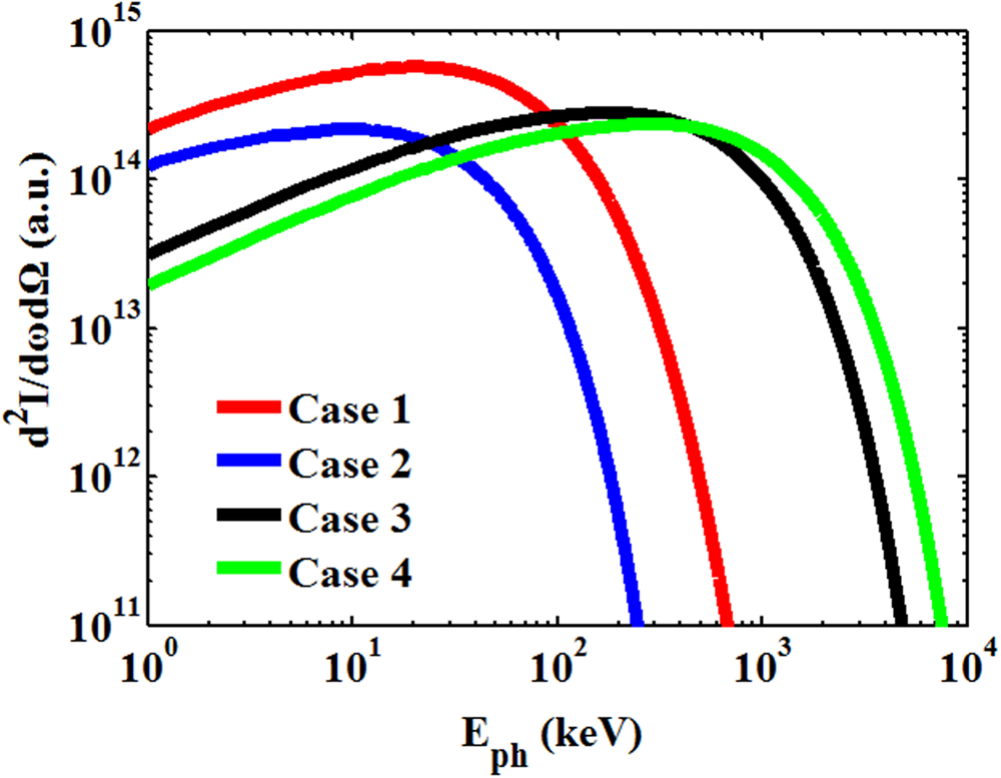
On-axis radiation spectra emitted by the randomly selected electrons in four different cases. (See Methods for the detailed calculation of the spectra and the critical photon energy of the radiation).

Usually, generation of high-energy betatron radiation from high-energy electron beams with large oscillation amplitudes is efficient. The on-axis radiation spectrum was calculated (see Methods) by integrating the contributions from four hundred randomly selected electrons in Fig. 2(g,h), considering the proportions of the electron numbers at the two different energy ranges. The numerical values in Fig. 2(a–h) are the total number of macroparticles. The same procedure was followed for the other three cases, and the spectra are shown in Fig. 3. For case 4, the radiation spectrum has a maximum critical photon energy of *E*_*c*_ ~ 820 keV. In contrast, case 1 corresponds to a critical photon energy of *E*_*c*_ ~ 75 keV.

## Discussion

**Figure 4:**
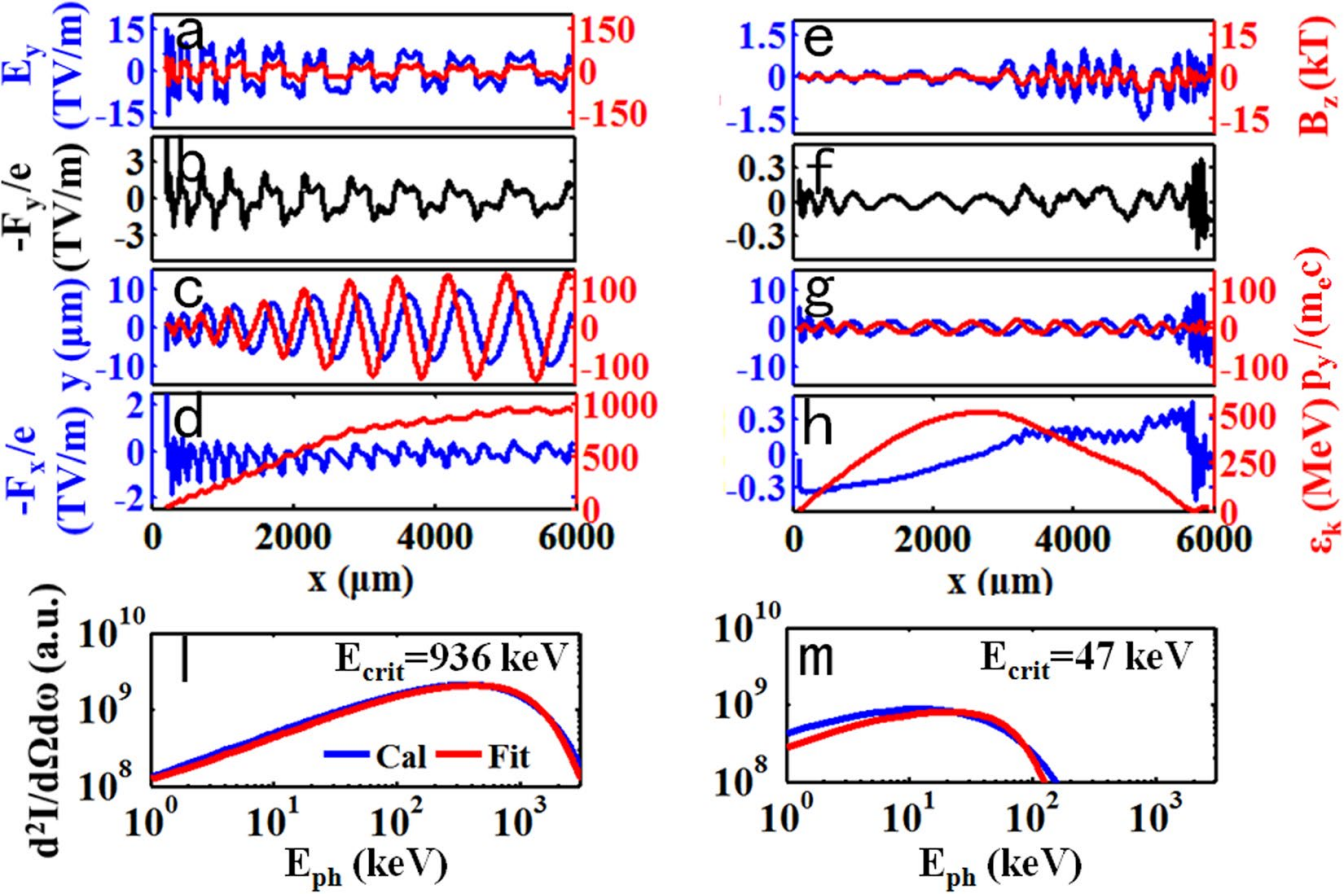
Behaviour and radiation of two tracked electrons in the second laser field (**a**–**d**,**l**) and the self-generated field (**e**–**h**,**m**). (**a**,**e**) are the transverse electric field E_y_ (blue line) and magnetic field B_z_ (red line) felt by the moving electron. (**b**,**f**) are the combined transverse force −F_y_/e, where F_y_ = q·E_y_ − q·v_x_·B_z_. (**c**,**g**) are the trajectory (blue line) and transverse momentum (red line) of the two electrons. (**d**,**h**) are the longitudinal acceleration field (blue line) and the energy evolution of the two electrons. (**l**) and (**m**) are the corresponding forward direction radiation spectra.

To discuss the influence of the second laser pulse on the dynamic behaviour of the accelerated electrons in the plasma bubble, two ionization-injected electrons located in and before the second laser field were tracked. The electromagnetic field felt by the two electrons is shown in Fig. 4(a,e). This result demonstrated that the intensity of the laser-driven electric/magnetic field was one order of magnitude higher than that of the self-generated field of the bubble. Moreover, the transverse momentum of the electron in the laser field was increased by one order of magnitude, as shown in Fig. 4(c,g). The dynamics of single electron interaction with the linearly polarized laser field and the self-generated field was analysed. Consider a linearly polarized plane wave with *E*_*ylaser*_ = *E*_0_ cos(*φ*) and *B*_*zlaser*_ = *E*_*ylaser*_/*v*_*ph*_, where *E*_0_ is the amplitude of the laser field, *φ* is the phase of the electric field, and *v*_*ph*_ = *ω*_0_/*k* is the phase velocity. In addition, the self-generated electromagnetic field of the plasma is written as *E*_*ys*_ and *B*_*zs*_. The transverse dynamic behaviour of the electron can be described by the momentum equation shown in eq. (1), where *v*_*x*_ is the electron speed along the laser propagation direction.

1 $$\frac{d{p}_{y}}{dt}=-\,e{E}_{0}(1-\frac{{v}_{x}}{{v}_{ph}})\cos (\phi )-e{E}_{ys}+e{v}_{x}{B}_{zs}$$

When the velocity of the electron is close to c, d*v*_*x*_/d*t* ~ 0 and d(1 − *v*_*x*_/*v*_*ph*_)/d*t* ~ 0, as *v*_*x*_ is a slow variable. The phase seen by the relativistic electron can be written as *ω*_*L*_ *t* = (1 − *v*_*x*_/*v*_*ph*_) *ω*_0_*t*. Therefore, eq. (1) can be simplified to eq. (2)^30^.

2 $$\frac{{d}^{2}{p}_{y}}{d{t}^{2}}+{\omega }_{\beta }^{2}{p}_{y}={m}_{e}c{a}_{0}{\omega }_{L}^{2}\,\sin ({\omega }_{L}t)$$

where *ω*_*β*_ is the betatron frequency and *a*_0_ = *eE*_0_*/m*_*e*_*ω*_0_*c*. Moreover, when the betatron frequency varies by roughly twice the laser frequency *ω*_*L*_, the electron transverse momentum will grow faster^31–34^, as shown in Fig. 4(c). The large electron oscillation amplitude and transverse momentum are mainly caused by electron betatron resonance with the laser field^30^.

Because the transverse wiggling force from the contribution of the laser field was approximately twenty times higher than that of the self-generated field of the bubble, as shown in Fig. 4(b,f), the electron oscillation amplitude in Fig. 4(c) could be up to ~10 μm, approximately one order of magnitude higher than that in the case shown in Fig. 4(g). The equivalent wiggler strength parameter was approximately 187, estimated by $K=e{B}_{0}{\lambda }_{\beta }/2\pi {m}_{e}c$ with *λ*_*β*_ ~ 0.5 mm and *B*_*0*_ ~ 4000 T, equivalently calculated by ${F}_{y}/(e\cdot c)$. This value was much higher than *K* ~ 11 for the case of the self-generated field. Because the magnetic field force *e·v*_*y*_*·B*_*z*_ was of comparable order to the accelerating electric field force, the accelerating field was modulated by the *e·v* × *B* force showing a dual frequency of the magnetic field, as shown in Fig. 4(d). Compared to the tracked electron before the second laser field, which experienced a smoother accelerating field, as shown in Fig. 4(h), when the electron caught up with the tail of the driven pulse at *x* ~ 3000 μm, modulation occurred. However, the electron also entered the deceleration field, and the oscillation amplitude did not increase at first. When the Doppler-downshifted frequency ${\omega }_{D}$, seen by the decelerated electron, satisfied the condition of ${\omega }_{D}=h\cdot {\omega }_{\beta }$^26^, where h is a harmonic number, the oscillation amplitude increased suddenly at *x* ~ 5500 μm. Therefore, possession of a high energy gain and a large amplitude simultaneously by these electrons was difficult. To obtain the radiation of the two electrons, we plugged the trajectories of the two electrons into the synchrotron radiation equation^24^ (see Methods). Figure 4(l) shows the on-axis radiation spectrum of the electron wiggling in the laser field with *E*_c_ = 936 keV, which was one order of magnitude higher than that in Fig. 4(m). The divergence in the direction of the oscillation could be given by K/γ^35^, and the divergences for the two tracked electrons were approximately 187/1800 ~ 100 mrad and 11/1000 ~ 10 mrad.

**Figure 5:**
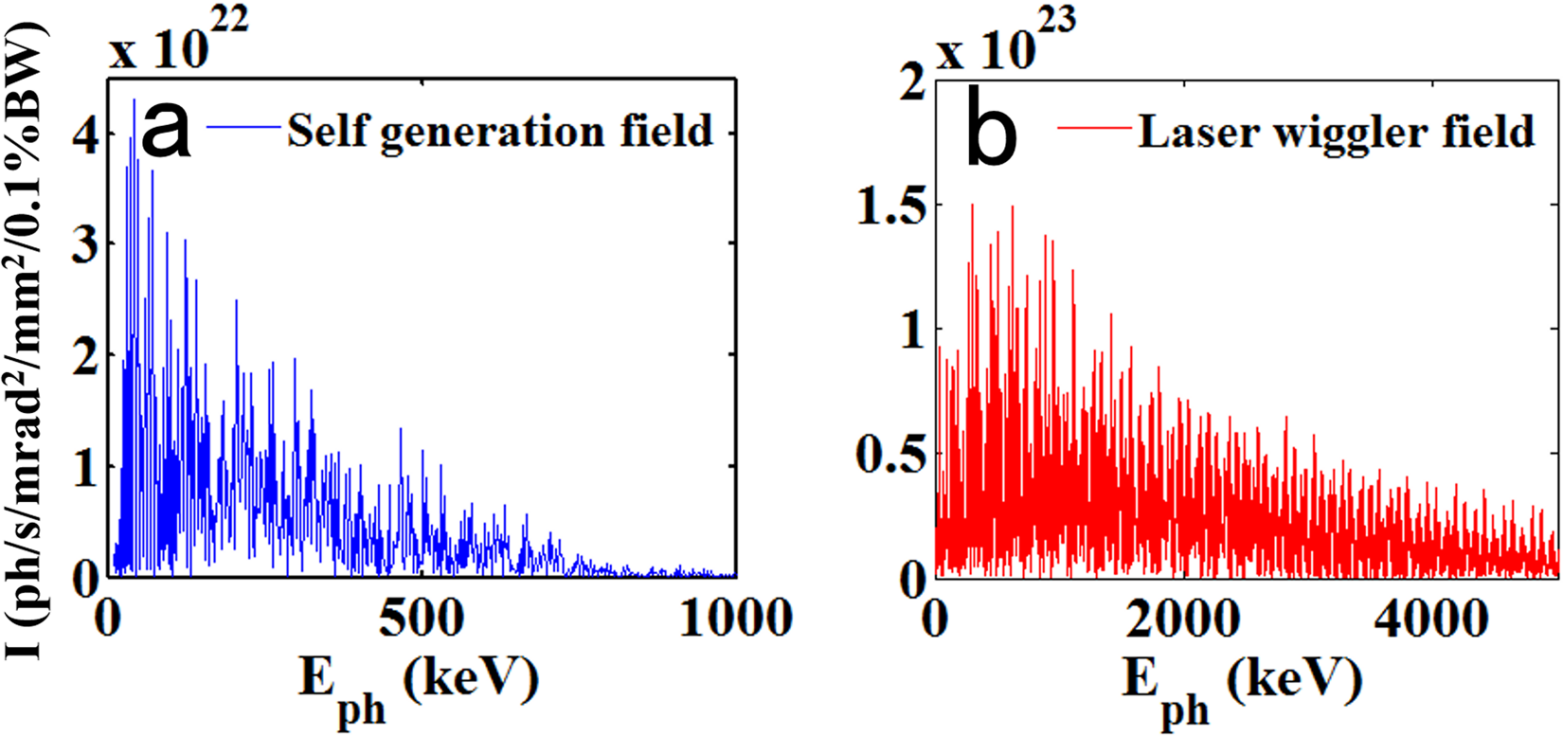
Radiation brightness of an electron beam wiggling in a self-generated field and an external laser field.

To estimate the radiation for the electron beam wiggling in the self-generated field and laser field, the electromagnetic field felt by the electron was taken as equivalent to the magnetic field of a periodic array of magnets. The two wiggler parameters used in the calculation were mentioned earlier, *K* = 11 and 187. The electron beam was conservatively estimated to have a peak energy of 750 MeV, an energy spread of 33.3%, a charge of 50 pC, and a beam length of 5 fs, in accordance with the PIC simulation and some existing experiment results^36–38^. The electron beam divergences were estimated to be 5 mrad and 10 mrad. By using the Sirepo code^39^, the synchrotron radiation from the wiggler was calculated and is shown in Fig. 5.

Figure 5(a) shows the radiation spectrum of the electron beam wiggling in the self-generated field of the bubble, and the peak brightness was approximately 4.5 × 10^22^ ph/s/mrad^2^/mm^2^/0.1%BW at 42 keV. In contrast, the peak brightness for the laser wiggler field could be up to 1.5 × 10^23^ ph/s/mrad^2^/mm^2^/0.1%BW at 627 keV and 1.2 × 10^23^ ph/s/mrad^2^/mm^2^/0.1%BW at 1 MeV, as shown in Fig. 5(b).

## Conclusion

In conclusion, a new method of gamma-ray generation from a wakefield-accelerated electron beam wiggling in an external laser field is presented. A laser pulse with a power of 100 TW drives the plasma wakefield, accelerating an electron beam in the regime of ionization injection, and is followed closely by a second 50 TW laser pulse. When the accelerating electrons experience the second laser field, the wiggling field is one order of magnitude higher than that for the self-generated field, and the equivalent strength parameter K ~ 187. The peak brightness of betatron radiation reaches 1.2 × 10^23^ ph/s/mrad^2^/mm^2^/0.1%BW at 1 MeV, which exceeds that of the third generation synchrotron light sources. Such an ultra-fast and brilliant gamma-ray source could be widely used in photonuclear reactions and for ultra-fast diagnosis of dense materials. In addition, because the betatron radiation emission from ionization injection has a tuneable polarization^40,41^, the second laser pulse directly forces the accelerated electron oscillation in the direction of laser polarization, which is propitious for generating high polarization ratio radiation. Such polarized gamma-ray emission could be applied to fields relying on magneto-optical phenomena.

## Methods

### PIC simulations

2D-PIC simulations were carried out using the KLAPS code^42,43^, and the tunnel-ionization model was adopted for field ionization. The simulation box size was 100 × 100 μm^2^ with 3000 × 2000 cells in the x and y directions, and one cell contained four macroparticles. The simulation box propagated along the x-axis at the speed of light. Moreover, a third-order time interpolation for the B field was used in this simulation^44^. The two p-polarized laser pulses had the same wavelength of 800 nm, and the spot size *w*_*0 = *_15 μm. The energy of the first laser was 3 J, and that of the second was 1.5 J; both were focused at 50 μm after the front edge of the plasma. The two pulses both had a Gaussian transverse profile and a sine-squared longitudinal envelope with a pulse duration FWHM of 30 fs. The neutral nitrogen longitudinal profile had a 100 μm up-ramp followed by a 6 mm long plateau with a uniform density of 6 × 10^16^ cm^−3^, and the background pre-ionized plasma density was 3 × 10^18^ cm^−3^.

### Single electron radiation calculations

The radiation of the electron beam was calculated by plugging the trajectories of the selected electrons into eq. (3) for the radiated energy per frequency and per solid angle^21^, where r(*t*) is the position of the electron, *β*(*t*) is the normalized velocity, $\dot{\beta }(t)$ is the normalized acceleration, n is the direction of observation, and *ω* is the emission frequency.

3 $$\frac{{d}^{2}I}{d\omega d{\rm{\Omega }}}=\frac{1}{4\pi {\varepsilon }_{0}}\frac{{e}^{2}}{4{\pi }^{2}c}\times {|{\int }_{-\infty }^{\infty }\frac{n\times \{[n-\dot{\beta }(t)]\times \beta (t)\}}{[1-\beta (t)\cdot n]}{e}^{i\omega (t-n\cdot {\rm{r}}(t)/c)}dt|}^{2}$$

However, for relativistic particles, essentially all the frequency spectra are at much higher frequencies ($\omega \gg (c/\rho )$), where *ρ* is the instantaneous curvature radius of the electron trajectory. Thus, the radiation can also be calculated with the approximate form of eq. (3), shown in eq. (4)^21^, where $\xi =\frac{\omega \rho }{3c}{(\frac{1}{{\gamma }^{2}}+{\theta }^{2})}^{2/3}$, *θ* is the observation angle, and ${K}_{1/3}(\xi )$ and ${K}_{2/3}(\xi )$ are modified Bessel function.

4 $$\frac{{d}^{2}I}{d\omega d{\rm{\Omega }}}=\frac{{e}^{2}}{3{\pi }^{2}c}{(\frac{\omega \rho }{c})}^{2}{(\frac{1}{{\gamma }^{2}}+{\theta }^{2})}^{2}[{K}_{2/3}^{2}(\xi )+\frac{{\theta }^{2}}{1/{\gamma }^{2}+{\theta }^{2}}{K}_{1/3}^{2}(\xi )]$$

Therefore, the on-axis radiation can be simplified by setting θ = 0, as shown in eq. (5).

5 $$\frac{{d}^{2}I}{d\omega d{\rm{\Omega }}}=\frac{{e}^{2}}{3{\pi }^{2}c{\gamma }^{{\rm{4}}}}{(\frac{\omega \rho }{c})}^{2}[{K}_{2/3}^{2}(\frac{\omega \rho }{3c{\gamma }^{4/3}})]$$

According to the electron trajectory, we can obtain the instantaneous curvature radius ρ. Taking ρ and the corresponding γ into eq. (5) and then summing all the data at different ω, the spectra were obtained, as shown in Figs 3 and 4(l,m). The critical photon energy was calculated by fitting the spectrum with the synchrotron radiation spectrum ${(E/{E}_{crit})}^{2}[{K}_{2/3}^{2}(E/{E}_{crit})]$.
